# Supplementary material for: Room-temperature chiral charge pumping in Dirac semimetals
Source: Nat Commun. 2017 Jan 9;8:13741. doi: 10.1038/ncomms13741 (PMC5227330; doi:10.1038/ncomms13741)
Supplement: Supplementary Information — Supplementary Figures 1-18, Supplementary Tables 1-2, Supplementary Notes 1-11 and Supplementary References [file ncomms13741-s1.pdf]

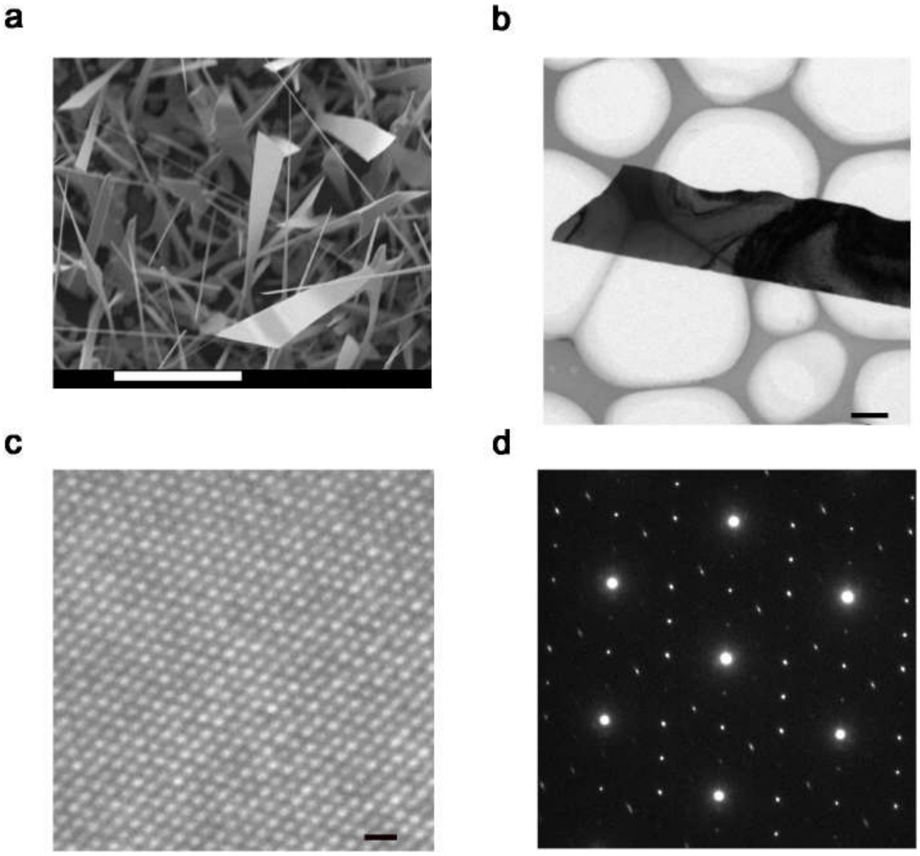

Supplementary Figure 1. Structural characterizations of Cd_3As_2 nanoplates. (**a**) A typical scanning electron microscopy image of as-grown Cd_3As_2 nanoplates. The white scale bar corresponds to 10 μm. (**b**) A transmission electron microscopy (TEM) image of a Cd_3As_2 nanoplate on a holey carbon grid. The black scale bar corresponds to 2 μm. (**c**) High-resolution TEM picture revealing a perfect crystalline structure. The black scale bar corresponds to 1 nm. (**d**) The corresponding selected area diffraction pattern.

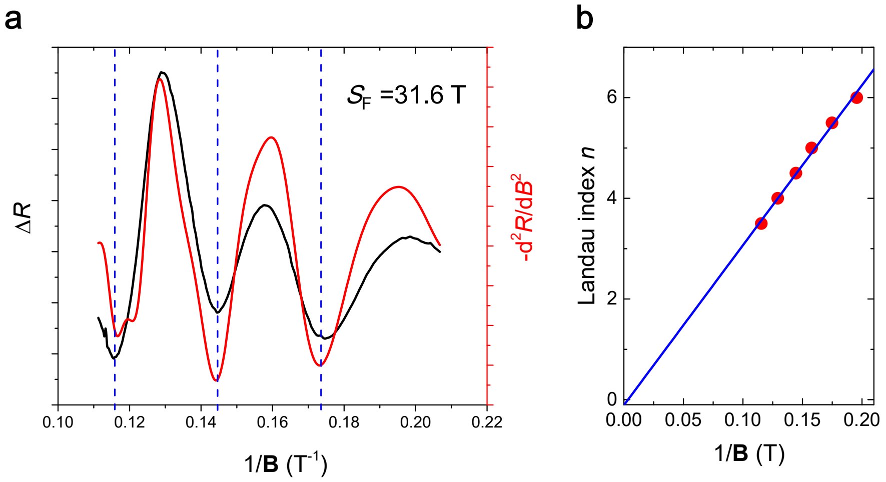

Supplementary Figure 2. Shubnikov-de Haas (SdH) oscillations in Sample N1. (**a**) SdH oscillations from both Δ*R* (the black curve) and the negative second derivative of magnetoresistance (MR) (the red curve). (**b**) The Landau fan diagram, revealing a nontrivial Berry phase.

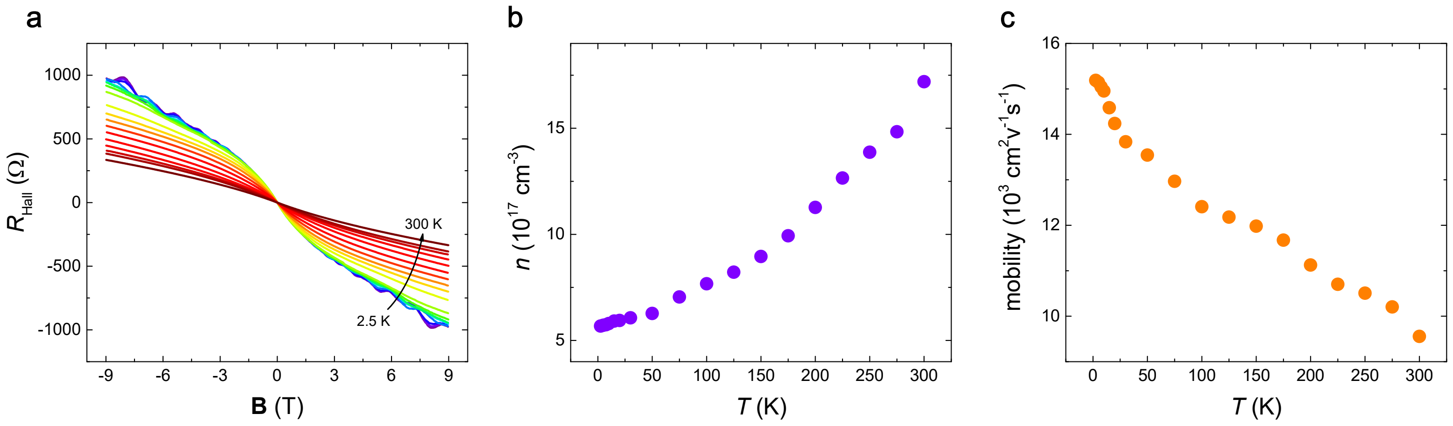

Supplementary Figure 3. Hall effect of sample N1. (**a**) Hall resistance at different temperatures. (**b-c**) Calculated carrier density (**b**) and mobility (**c**) as a function of temperature from Hall effect measurements.

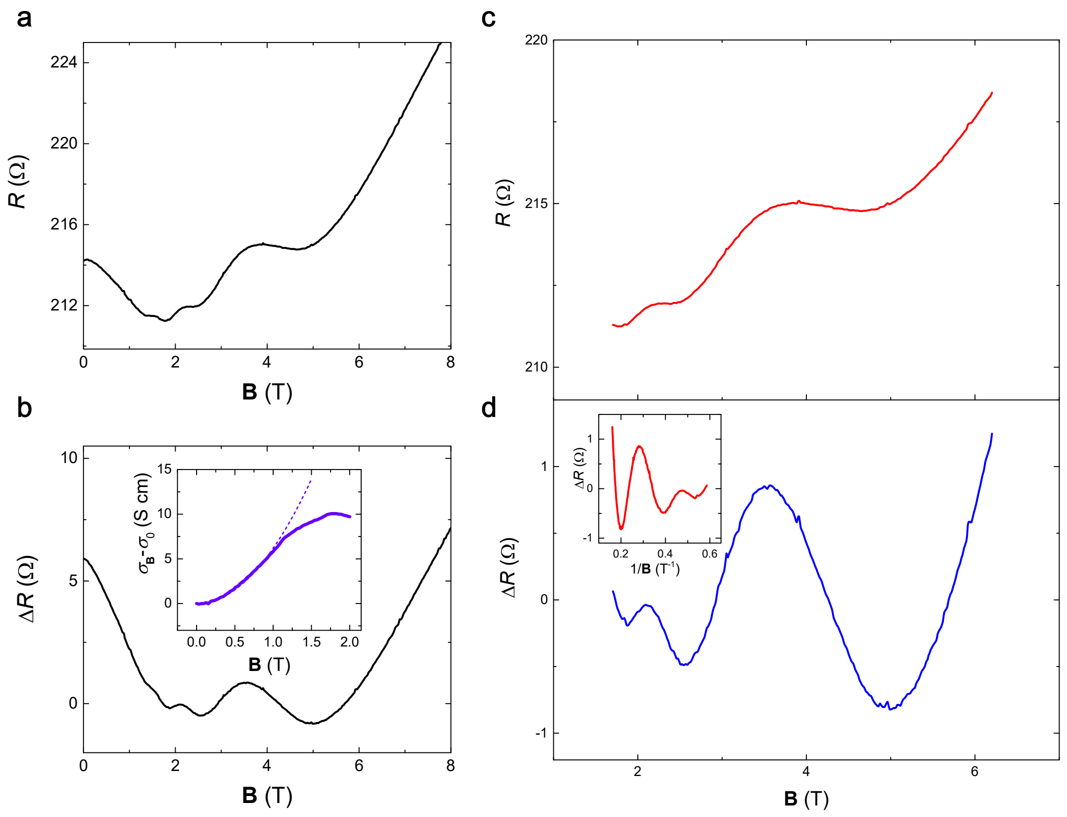

Supplementary Figure 4. Analysis of the negative MR and the SdH quantum oscillations in sample N1. (**a**) The original longitudinal MR curve at 2.5 K, showing a negative MR below 2 T. (**b**) The MR data after extracting a quadratic background. The inset is a quadratic fitting to the low field positive magneto-conductance change σ_B-σ_0. (**c-d**) The original MR data (**c**) and pure oscillations (**d**) with background removed under parallel magnetic field, respectively. The inset in **d** is ΔR plotted against 1/**B**. The oscillations show a periodic behavior with 1/**B**, implying that the minima around 5 T is actually due to the SdH oscillations.

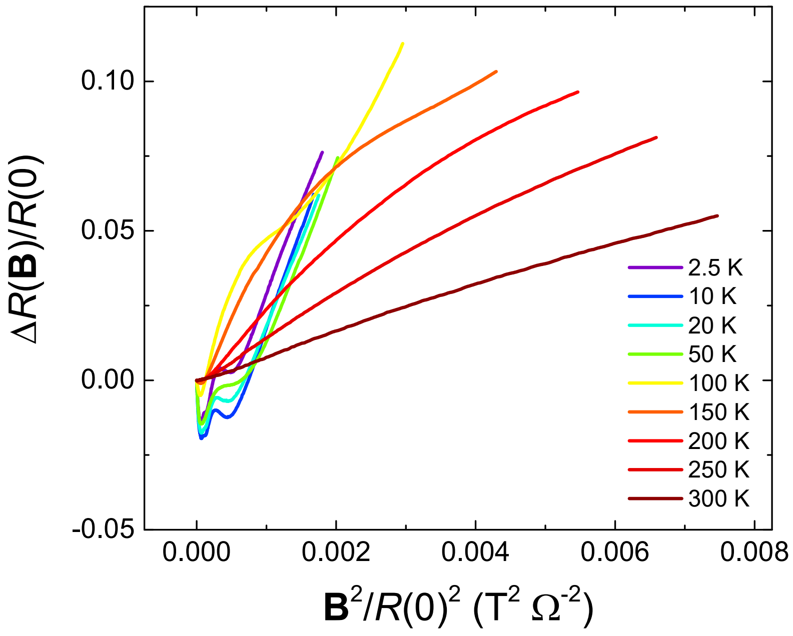

Supplementary Figure 5. The Kohler's plots of the MR curves in sample N1. Anomalous behavior is observed at the low field regime at low temperatures, where the negative MR occurs.

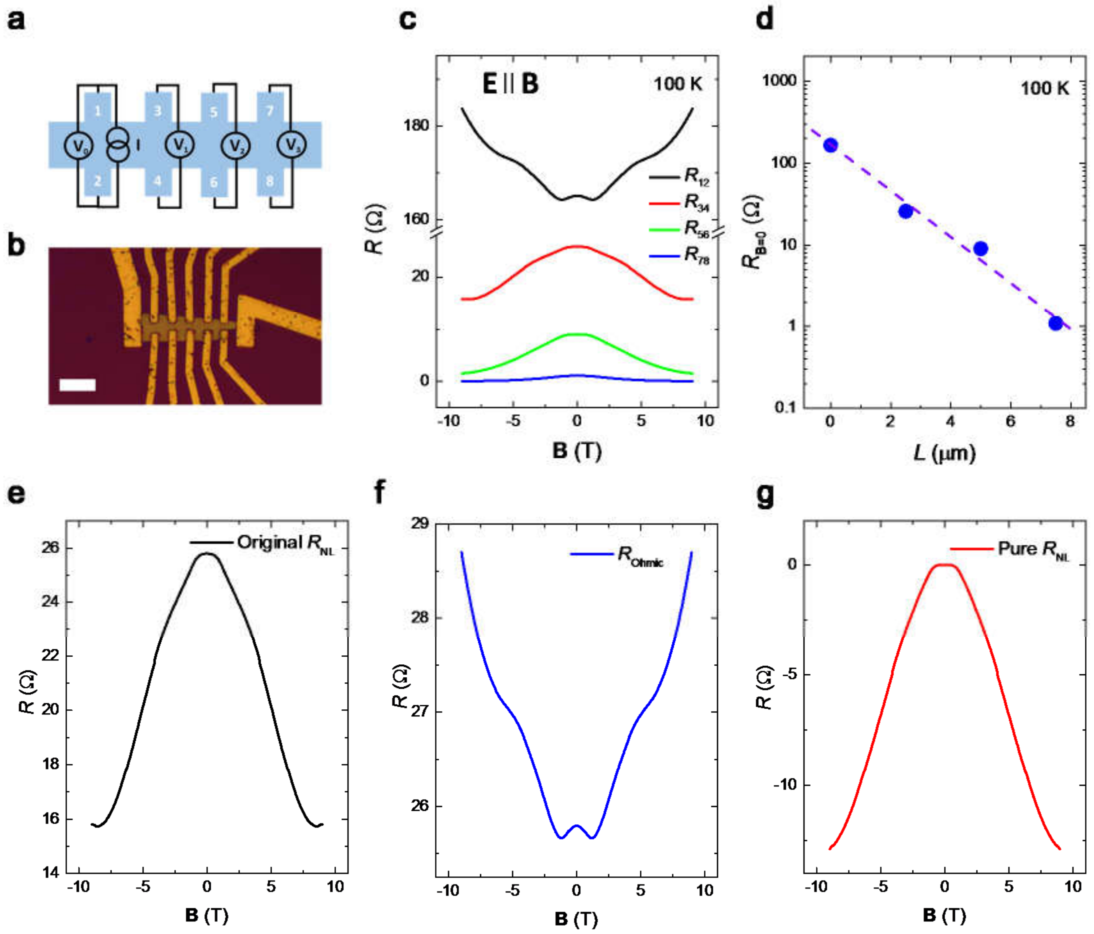

Supplementary Figure 6. Ohmic contribution in nonlocal transport in sample N1. (**a-b**) The schematic view (**a)** and optical picture (**b**) of the Cd_3As_2 nanoplate device with several pairs of Hall electrodes. The white scale bar in **b** corresponds to 2 μm. (**c**) Originally measured nonlocal resistance R_{NL} ($R_{34}, R_{56},$ and R_{78}) and local resistance R_{L} (R_{12}). R_{L} and R_{NL}, showing opposite ***B***-dependence. (**d**) The zero field resistance, following an exponential decay with L. (**e-g**) A direct comparison of original nonlocal resistance (**e**), calculated Ohmic contribution (**f**), and pure nonlocal resistance (**g**).

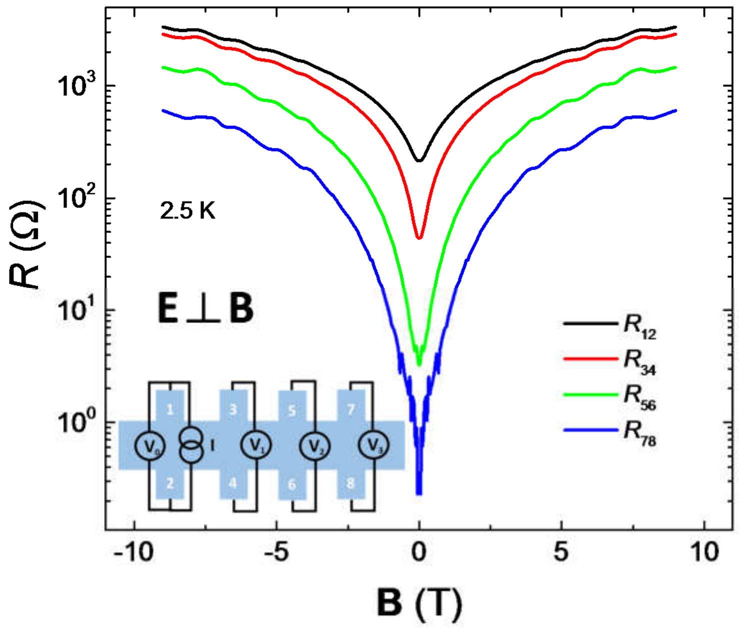

Supplementary Figure 7. Pure Ohmic resistance in nonlocal transport in sample N1. The detected pure Ohmic resistance is achieved by eliminating the valley transport under the perpendicular electric and magnetic fields. The nonlocal resistance here follows an exponential decay with L at all field range.

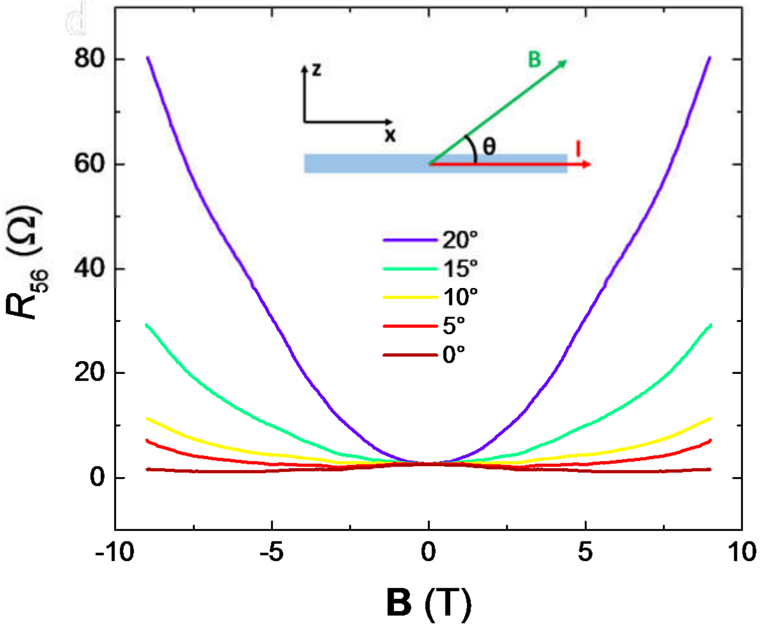

Supplementary Figure 8. Angular dependence of nonlocal resistance R_{56} in sample N1 when tilting B at 10 K. θ is defined as the angle between the current and $\boldsymbol{B}$. The negative nonlocal resistance decreases rapidly when the magnetic field is tilted towards out-of-plane.

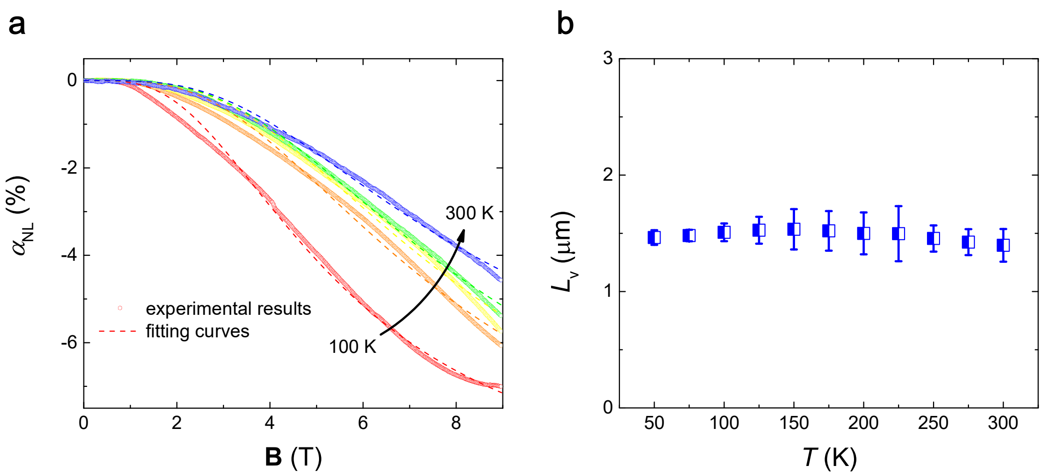

Supplementary Figure 9. Fitting curves and calculated parameters of the nonlocal transport in sample N1. (**a**) Fittings of the α_{NL}-$\boldsymbol{B}$ curves at different temperatures. The field dependence of nonlocal ratio can be well fitted. (**b**) Calculated L_v at different temperatures. The value from the fitting of field dependence is similar to that extracted from the length dependence shown in Fig. 5 in the main text. The error bar is estimated based on the fitting errors.

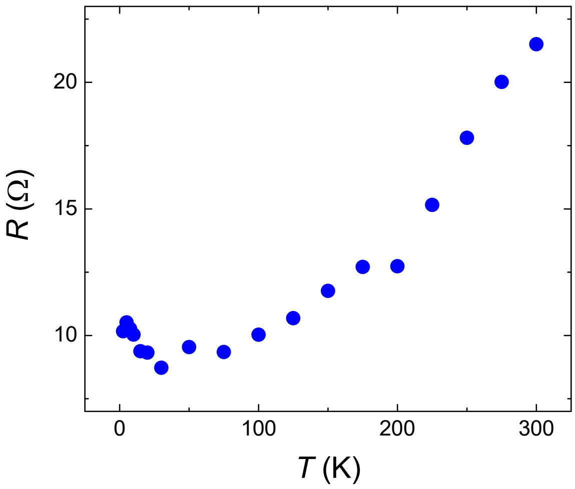

Supplementary Figure 10. The contact resistance at different temperatures in sample N1. The temperature dependence of contact resistance agrees with the trend of nonlocal ratio with temperature.

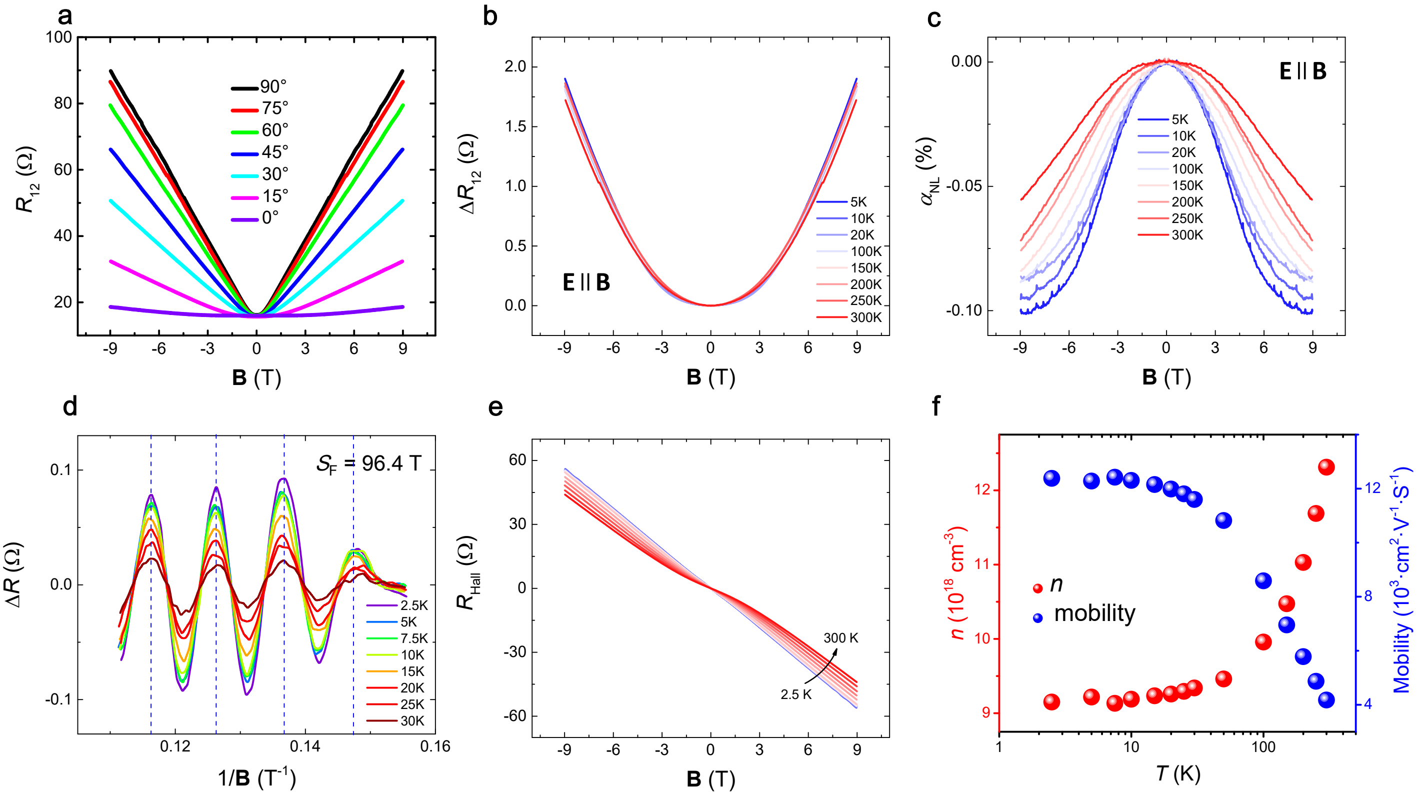

Supplementary Figure 11. Nonlocal transport of in sample N3. (**a**) The angular dependence of R_L-**B** curves with θ defined as the angle between the applied current and the magnetic field. $\theta = 90°$ represents that **B** is perpendicular to the sample. (**b**) $\Delta R_L = R_L(\mathbf{B},\ T) - R_L(0,\ T)$ measured in a two-terminal configuration with $\theta = 0°$. (**c**) Measured nonlocal response α_{NL}-**B** curves at different temperatures with α_{NL} being the ratio between R_{NL} and R_L. (**d**) Extracted SdH oscillation curves, showing a uniform periodicity against 1/**B**. (**e**) Hall data of the sample when $\theta = 90°$. (f) The calculated carrier density n and mobility based on Hall effect.

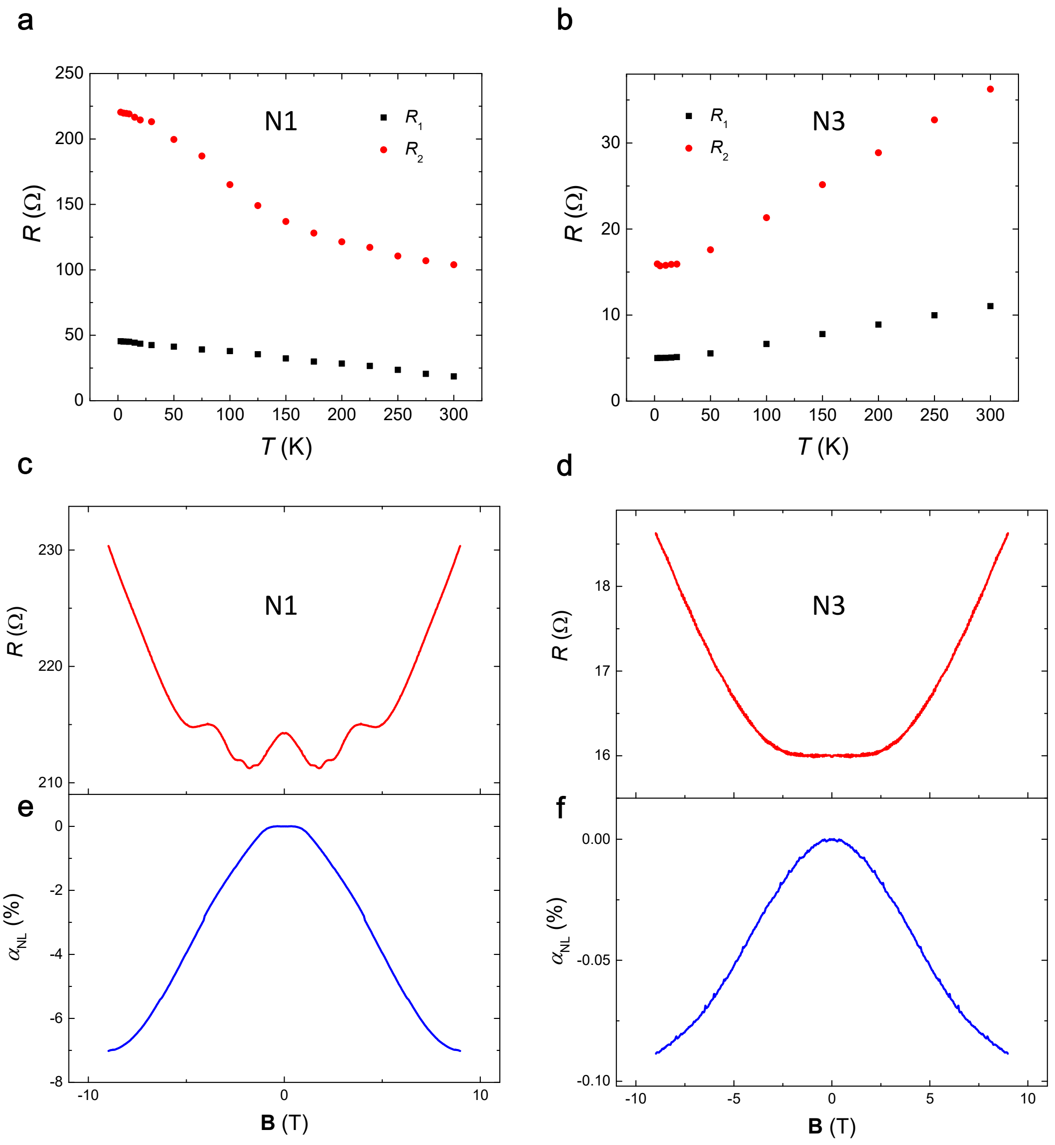

Supplementary Figure 12. Comparison of samples N1 and N3 with different Fermi levels. (a-b) The temperature dependent resistance with current parallel (R_1) and perpendicular (R_2) to the [110] crystal direction in samples N1 and N3, respectively. (**c-d**) The local resistance at 2.5 K for samples N1 and N3, respectively. (**e-f**) The nonlocal ratio at 100 K for samples N1 and N3, respectively. The nonlocal ratio is extracted at 100 K due to the development of bad contacts in high resistivity samples at lower temperatures.

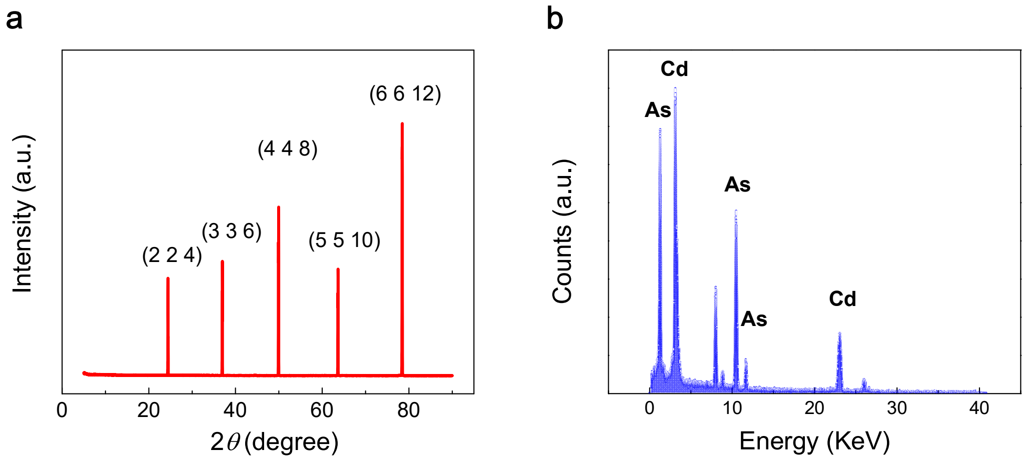

Supplementary Figure 13. Material characterizations of Cd_3As_2 bulk crystal. (**a**) A typical X-ray diffraction patterns of the single crystal Cd_3As_2 with (112) surface. (**b**) A typical energy dispersive X-ray spectrum of the crystal, showing the atomic ratio of Cd:As=3:2. The rest peaks are originated from the system background and not matched with magnetic elements like Fe or Cr.

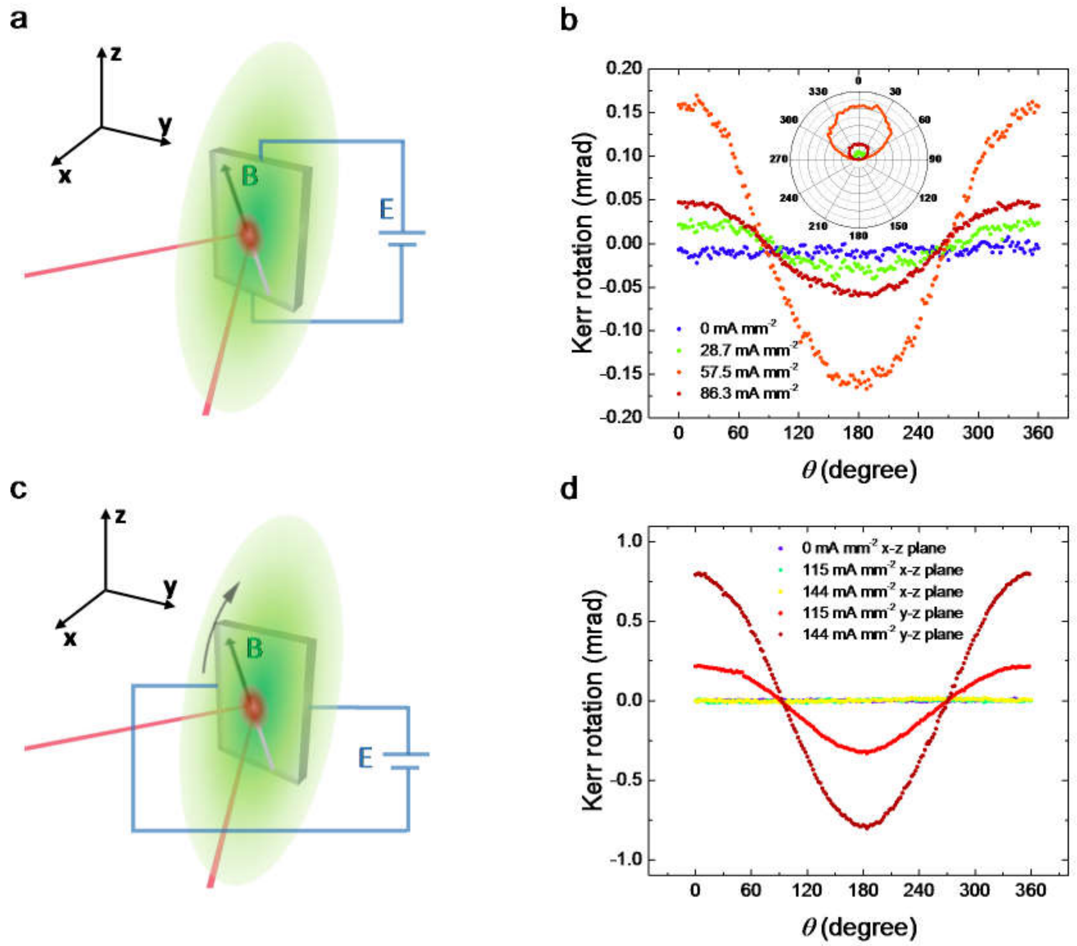

Supplementary Figure 14. Comparisons of the rotational magneto-optical Kerr effect (ROT-MOKE) of Cd_3As_2. (**a**) Schematic ROT-MOKE I set-up. The magnetic field ***B*** is rotated in the x-z plane. A constant electric current was applied in the **B**-rotating plane. The laser is horizontally polarized with a wavelength of 670 nm. (**b**) ROT-MOKE signals of Cd_3As_2 crystal under different current density, adopting a cosine-function dependence on θ. Here θ is defined as the angle between the electric and magnetic field. For (**b**) the magnetic field is fixed at 2000 Oe. The insets are data plotted in polar coordinates. (**c**) Schematic view of the ROT-MOKE II set-up. The only difference between ROT-MOKE I and II is the direction of the electric bias. Here, **E** is perpendicular to **B**-rotating plane. (**d**) Comparison of the ROT-MOKE data from the main text with the data from the ROT-MOKE II set-up. The magnetic field was fixed at 2000 Oe. No Kerr rotation was observed in the ROT-MOKE II configuration.

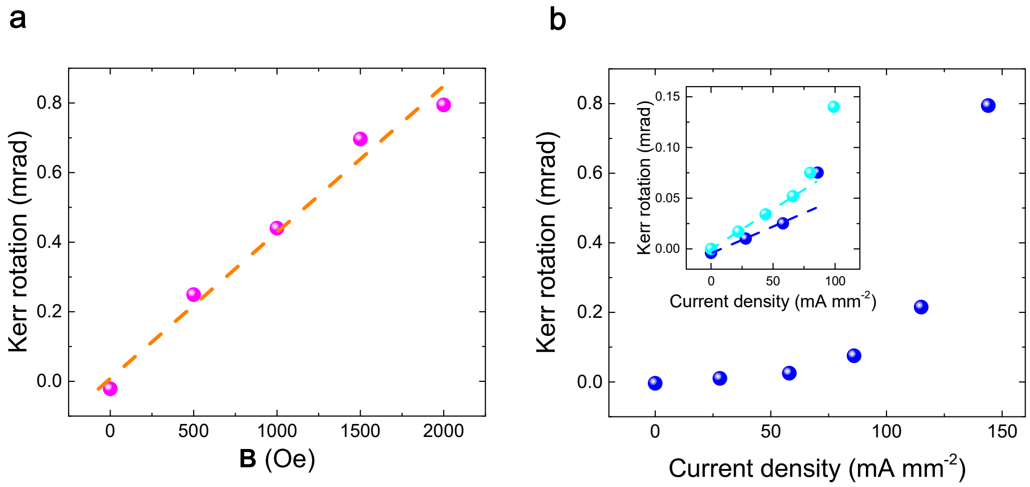

Supplementary Figure 15. The relationship of Kerr rotation with magnetic field (a) and current density (b). The Kerr rotation value is selected from the peak value in the ROT-MOKE curve. For **a**, the magnetic field is in y-z plane (Fig. 6 in the main text) and the current density is 144 mA mm^{-2}. For **b**, the magnetic field is 2000 Oe and parallel with electric field. The inset of **b** is an enlarged view of the Kerr rotation (blue dots) in the low current density regime, showing linear relationship with current density. A strong deviation from linear relationship is observed in the large current density regime. Such behavior is also confirmed by the second sample (cyan dots) we measured.

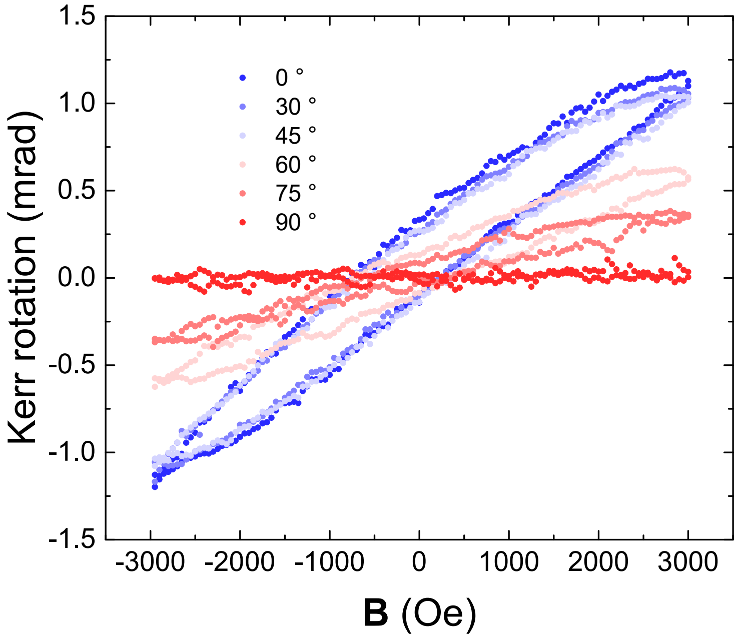

Supplementary Figure 16. MOKE data of Cd_3As_2 bulk crystal. MOKE signals of Cd_3As_2 crystal at different magnetic field directions. The measurements adopt the geometry from the main text (Fig. 1b).

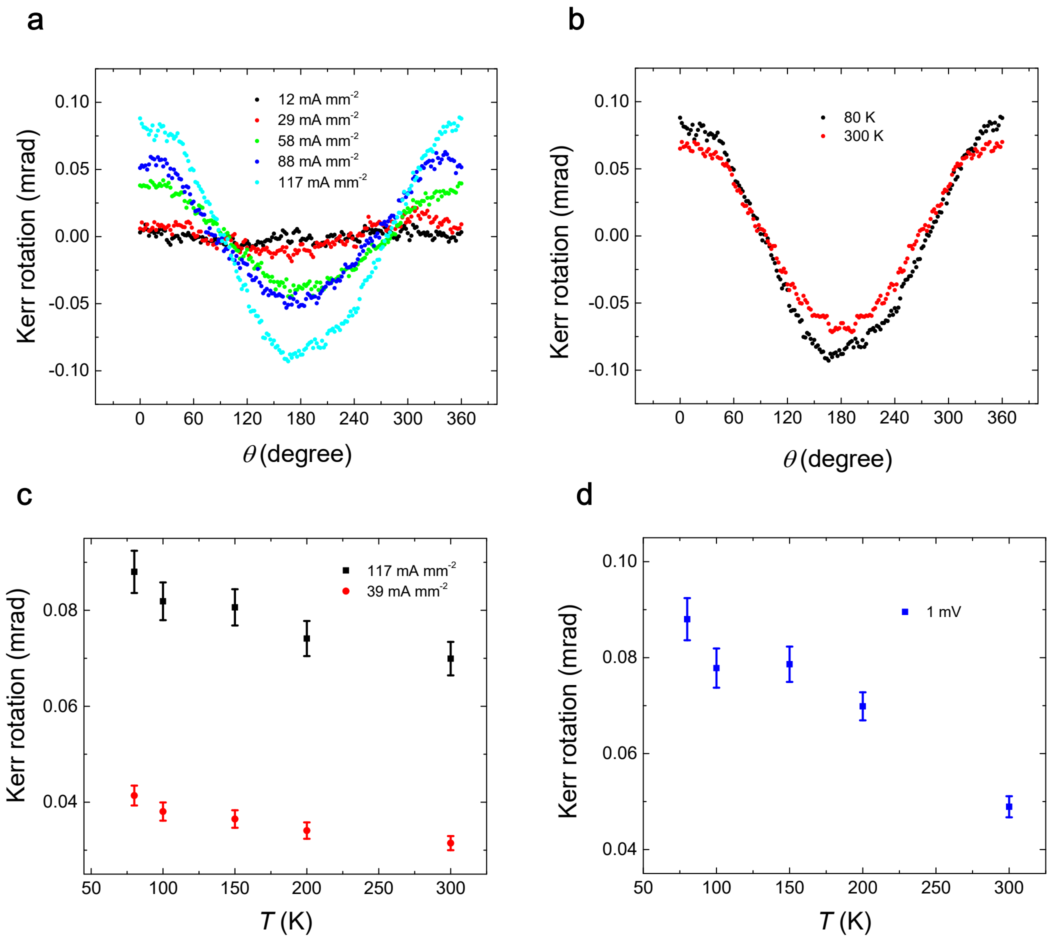

Supplementary Figure 17. Temperature-dependent ROT-MOKE signals at different temperatures. (**a**) ROT-MOKE signals of Cd_3As_2 crystal under different current density at 80 K, adopting a cosine-function dependence on θ. Here θ is defined as the angle between the electric and magnetic field. The magnetic field is 1000 Oe. (**b**) Comparison of the ROT-MOKE data at different temperatures with the same current density of 117 mA mm^{-2}. (**c**) Kerr rotation at different temperatures with different current density at 1000 Oe. (**d**) Kerr rotation at different temperatures with a fixed bias voltage of 1 mV. Note that this voltage is the actual voltage drop on the sample. The error bars are estimated as five percent of the signal value.

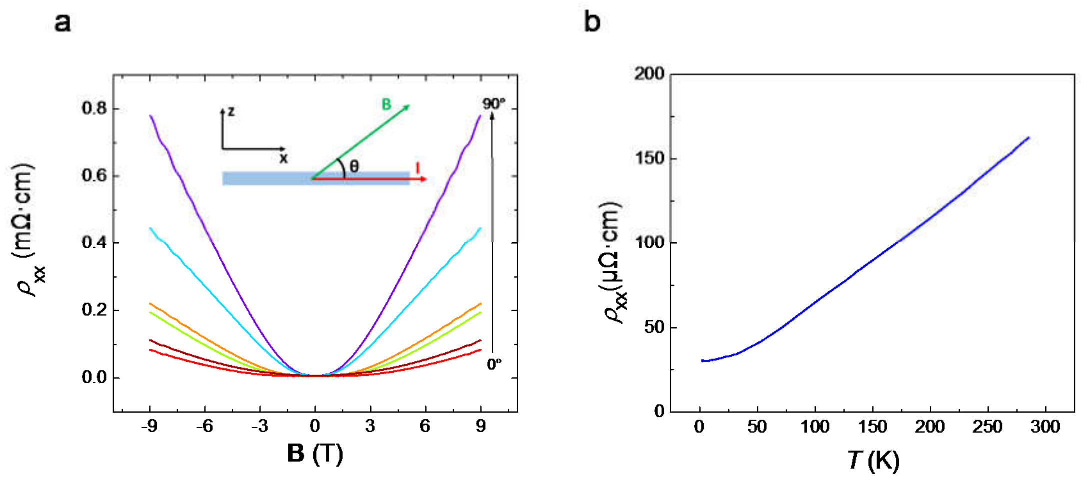

Supplementary Figure 18. Electrical transport properties of Cd_3As_2 bulk crystal. (**a**) The angular dependence of longitudinal resistivity at 3 K. The SdH oscillations are observed at different angles. (**b**) The zero-field resistivity as a function of temperature.

Reference	Material	$\mathbf{B}_c$ (T)	T_c (K)	Mobility ($cm^2V^{-1}s^{-1}$)	MR ratio	E_F (meV)	n (10^{18} cm^{-3})
Xiong et al.[1]	Na_3Bi	5	100	2600	4.5	29	0.14
Li et al.[2]	$ZrTe_5$	~8	100	/	~70	Close to Dirac point	/
Huang et al.[3]	TaAs	5	50	163000	800	/	8.23
Zhang et al.[4]	TaAs	0.5	30	~400000	~6000	/	0.8
Yang et al.[5]	NbAs	3.5	50	245000	~110	/	/
Hirschberger et al.[6]	GdPtBi	~14	150	1500	~2	/	2
Li et al.[7]	Cd_3As_2	3-7	>300	/	~0.8	~54	~0.1
Li et al.[8]	Cd_3As_2	9	>300	~11000	1	/	0.022
This work	Cd_3As_2	2	150	15200	15	/	0.57

Supplementary Table 1. Summary of the physical parameters of topological semimetals with negative MR. The critical field $\mathbf{B}_c$ and critical temperature T_c of negative MR, mobility, transverse MR ratio at 9T, Fermi level E_F, carrier density n are provided. Strong negative MR tends to arise in systems with low carrier density and small transverse MR ratio.

Sample	n/cm^{-3}
N1	5.7×10^{17}
N2	4.6×10^{17}
N3	9.1×10^{18}

Supplementary Table 2. Comparison of carrier density in three samples. Samples with low carrier density tend to give strong chiral anomaly effect (evident by the nonlocal ratio and negative MR).

Supplementary Note 1. Structural characterizations of Cd_3As_2 nanoplates

The crystal structures of the synthesized products were characterized by X-ray diffraction (XRD), recorded on an X-ray diffractometer equipped with graphite monochromatized, Cu Kα radiation (λ = 1.5418 Å). The morphological, structural, and chemical characteristics of the synthesized products were investigated by scanning electron microscopy (SEM, JEOL 7800 and 7001) and transmission electron microscopy (TEM, FEI F20) equipped with energy-dispersive X-ray spectroscopy (EDS).

Supplementary Note 2. Analysis of magnetotransport data in Cd_3As_2 nanoplates

Quantum oscillations can give detailed information of the carrier and Fermi surface. During the transport measurements, clear SdH oscillations were resolved at low temperatures owing to the high mobility of the Cd_3As_2 nanoplates. The SdH oscillations of sample N1 are provided in Supplementary Fig. 2. In transport experiments, the phase factor γ can be obtained from the offset in the Landau fan diagram. According to the Lifshitz-Onsager quantization rule $S_{\mathrm{F}}\frac{\hbar}{eB}=2\pi(n-\delta+\gamma)$, the phase factor γgives the Berry phase ϕ_{B} by $\gamma=\frac{1}{2}-\frac{\phi_{\mathrm{B}}}{2\pi}$, where δ is an additional phase shift from the curvature of the FS, $\hbar$ is the reduced Planck's constant and e is the elementary charge. If using oscillation peak as integer Landau index, the phase factor is 0 for nontrivial case and 0.5 for trivial case. Supplementary Fig. 2b depicts the Landau fan diagram of Cd_3As_2, where the peak of oscillation denotes the integer Landau index. A nontrivial Berry phase is obtained. Hall effect in Supplementary Fig. 3a can be used to extract transport parameters such as carrier density (Supplementary Fig. 3b) and mobility (Supplementary Fig. 3c). Chiral magnetic effect can be significantly enhanced in the system with Fermi level close to the Weyl nodes.[9, 10] The low carrier density of samples N1 (Supplementary Fig. 3b) enables us to observe the negative magnetoresistance which was not accessible in previous studies with high-carrier-density samples.

As shown in Supplementary Figs 4 a and b, the negative MR emerges below 2 T and becomes overwhelmed by the positive MR background at higher fields. SdH oscillations start at about 1.5 T. The oscillation amplitude should increase with the magnetic field and be periodic with 1/**B** (refer to Supplementary. Fig 4c). The sudden drop of MR starting at 0 T is clearly not consistent with the conventional SdH oscillations that should vanish at zero field. The weak localization and disorder may also lead to the negative MR. However, the localization induced negative MR in disordered low-dimensional metals is typically restricted within several hundreds of Oe.[11, 12] In order to achieve negative MR within 1.5 T by localization, a very short

localization coherent loop is required, which is not consistent with the high mobility (over 10000 cm^2/Vs) in the sample. The interference in low-dimensional metal usually takes place within the 2D plane. So only magnetic field from out-of-plane will contribute to the self-intersecting scattering paths.[11] The negative MR in our study is observed in the in-plane fields. Meanwhile, Cd_3As_2 is a strong spin-orbit coupled system with a large *g* factor.[13] Thus, even if this kind of quantum interference affects the transport, it is more likely to be weak anti-localization instead of weak localization. It means that the localization from low dimension does not dominate the transport. Also, the negative MR was found to persist up to 150 K, much higher than the conventional localization behavior which requires a long phase coherence length. Another unique feature of the longitudinal MR is that a small variation of magnetic-field direction by 3 degrees will eliminate the negative MR (refer to Fig. 1c in the main text). The negative MR only appears when **E** and **B** is parallel. This is consistent with recent studies on negative MR observed in TaAs[3, 4] and $ZrTe_5$[2] bulk crystals. It is worth mentioning that the Weyl semimetal TaAs family also possesses the properties of ultrahigh mobility and large MR, which is very similar to Cd_3As_2[3, 4]. The $\mathbf{E} \cdot \mathbf{B}$ locking feature is a strong hint for the chiral magnetic effect as pointed out previously[1].

Cd_3As_2 has been proved to be single band conduction near the Fermi level.[13, 14] Thus, a linear scaling relation should be expected in the Kohler's plots for a system with constant scattering time over the Fermi surfaces at a fixed temperature. However, as shown in Supplementary Fig. 5, anomalous behavior is observed at the low field regime at low temperatures, where the negative MR occurs. The presence of strong conductance correction from chiral anomaly will lead to the violation of the Kohler's rule.

The temperature dependence of nonlocal transport and Kerr effect is different from that of negative magnetoresistance (MR). The reason for this discordance is that the longitudinal MR is a mixture of axial current and original background of resistance change under magnetic field. Topological semimetals, such as TaAs and Cd_3As_2, usually possess exceptionally high carrier mobility up to 10^5~10^7 $cm^2V^{-1}s^{-1}$.[3, 4, 14, 15] Due

to the high mobility and resultant long transport life time, dramatic change on resistivity can be induced by the application of magnetic field. Another evidence for the large background in MR is the requirement of perfect alignment of current and magnetic field direction. As shown in this study and previous reports[1], a small misalignment of the magnetic field will largely influence or even destroy the negative MR. The reported angle range of negative MR is much smaller than what is expected from the $\mathbf{E}\cdot\mathbf{B}$ field.[1] A large positive MR background may result in such behavior by overwhelming the contribution of chiral anomaly. In experiment, it's very hard to separate these two contributions. Thus the negative MR may not accurately reflect the real property of chiral anomaly.

In comparison, the background of nonlocal resistance or Kerr effect, as will be discussed below, can be either well subtracted or negligible. In the well-defined device geometry shown in Fig. 3b, $R_{\mathrm{Ohmic}} = (R\frac{x_2}{x_1})\frac{x_2}{\pi L}e^{-\pi L/x_2}$. Here R is the local resistance, L is the diffusion length, $(R\frac{x_2}{x_1})$ is the normalized effective local resistance for terminal 5-6. Thus, the background of nonlocal resistance (Ohmic resistance at nonlocal terminals) can be largely diminished through the control of the device geometry (the ratio of diffusion channel length over width). Furthermore, the residual Ohmic resistance can be easily subtracted based on the equation of R_{Ohmic} above. As for the Kerr effect, the control experiment of rotational magneto-optical Kerr effect (ROT-MOKE) at zero electric or magnetic field showed zero Kerr rotation at all angles. It excludes the existence of conventional Kerr effect originating from ferromagnetism or birefringence effect in Cd_3As_2. The detected Kerr signal should be only contributed by the chiral anomaly. Therefore, the negative MR adopts a different temperature dependence (or critical temperature) from the Kerr effect or nonlocal transport.

Supplementary Table 1 is a summary of the critical field $\mathbf{B}_c$, critical temperature T_c of negative MR and other transport parameters in various topological semimetals at low temperature. Since it is hard to evaluate the conventional longitudinal MR in this case, we used the transverse MR ratio for simplicity. The carrier density is also used as an

indication for the Fermi level. We can easily find out from the table that strong negative MR tends to arise in systems with low carrier density and small MR ratio (due to the comparably low mobility).

Supplementary Note 3. The effect of current jetting

As found in many high mobility systems, a magnetic-field-induced steering of the current may lead to an anomalous current density distribution and a negative longitudinal MR in the four-terminal contact geometry when the contacts are smaller than the cross-section of the sample.[4, 16, 17, 18] Recent studies also show that this effect may strongly affect the reliability of negative MR as evidence for the chiral anomaly.[18] For single crystals with extremely low resistance in which the four-terminal contact is necessary, one may distinguish the current jetting and the chiral-anomaly-induced negative MR by making contacts on both sides of the sample. The current jetting effect will give a suppressed and an enhanced MR at each side as shown in the study of Weyl semimetal TaP.[18]

Here in our case, the current jetting effect will not affect the analysis of both the negative MR and the nonlocal transport. In the negative MR study, we can observe the negative MR in both two-terminal and four-terminal contact geometries. The data in Fig.1 b and c is measured in a two-terminal geometry as a local resistance R_{12} (refer to Fig. 4 a and b for contact geometry and device picture). In two-terminal contact geometry, the current jetting effect does not affect the MR behavior. Here, since the Cd_3As_2 nanoplates are metallic and have a resistance ranging from 20 to 1000 Ω at zero field, the contact resistance does not strongly influence the magnetotransport measurements. As for the nonlocal transport, we have measured the nonlocal voltage at both sides (terminals 3-4 and 5-6 in Fig. 3b) of the local resistance terminal 1-2. Both the nonlocal MR show negative trend instead of opposite trends as the four-terminal contact study in TaP. Thus we can safely exclude the current jetting as the origin for the observed negative MR and nonlocal resistance.

Supplementary Note 4. Extracting Ohmic resistance

In order to extract the stray charge currents from the detected nonlocal signals, we use van der Pauw formula $R_{\text{Ohmic}} = \frac{\rho}{\pi} e^{-\pi L/W}$ to calculate the Ohmic resistance [19, 20], where resistivity $\rho = R_L W/L$. R_{L} is the longitudinal resistance. W and L are defined in Fig. 3a in the main text. From the Hall effect measurements in Supplementary Fig. 3, we know that the device hasn't reached the ballistic transport regime especially at high temperatures. Hence, the van der Pauw formula derived from the Poisson equation is still valid. In the well-defined device geometry shown in Fig. 3b, $R_{\text{Ohmic}} = R\frac{x_1}{\pi L} e^{-\pi L/x_1}$ and $R_{\text{valley}} = -k_{34} R e^{-L/L_v}$ for terminal 3-4. And for terminal 5-6, $R_{\text{Ohmic}} = (R\frac{x_2}{x_1})\frac{x_2}{\pi L} e^{-\pi L/x_2}$ and $R_{\text{valley}} = -k_{56}(R\frac{x_2}{x_1}) e^{-L/L_v}$. Here R is the local resistance, L_v is the valley diffusion length, $(R\frac{x_2}{x_1})$ is the normalized effective local resistance for terminal 5-6, and $k = \left(\frac{1}{\delta/B+1}\right)^2$ is a dimensionless coefficient determined by magnetic field and metal contact conductance.

Supplementary Fig. 6c is the original data for the detected nonlocal resistance of sample N1 in Fig. 4, which has a mixture of the valley diffusion and Ohmic resistance. By plotting the zero field resistance which has pure Ohmic contribution, we find that it also adopts an exponential decay with L. Supplementary Figs 6 e-g are the direct comparison of original nonlocal resistance, calculated Ohmic contribution, and pure nonlocal resistance. Moreover, we measured the nonlocal resistance under a perpendicular magnetic field (Supplementary Fig. 7). Under this condition, the detected nonlocal resistance adopts an identical field dependence in semi-log plot. It implies that it is reasonable to apply the van der Pauw formula for Ohmic resistance in our study.

Supplementary Note 5. Angular dependence of nonlocal resistance

Supplementary Fig. 8 shows the angular dependence of nonlocal resistance R_{56} (sample N1, without subtracting the Ohmic diffusion). The negative nonlocal resistance decreases rapidly when the magnetic field is tilted towards out-of-plane. This phenomenon is actually understandable. Unlike the original theoretical proposal, here we only use one magnetic field **B** to act as both generating and detecting field. Therefore, when rotating the direction of **B**, it leads to dual effects on the final nonlocal voltage. In the generating part, tilting **B** will suppress the induced valley polarization. And in the detecting part, tilting **B** will make the converted nonlocal voltage smaller. On the other hand, the MR ratio in Cd_3As_2 will be strongly enhanced when **B** is tilted out of plane as shown in previous papers, which may further overwhelm the negative **B**-dependence of nonlocal voltage by valley diffusion.

Supplementary Note 6. Valley-relaxation length from field dependence of nonlocal ratio

Valley-relaxation length L_v can be also fitted from the magnetic field dependence of the nonlocal ratio α_{NL}. The nonlocal response arising from the valley diffusion is given by [9]

$$\alpha_{NL} = \frac{R_{NL}}{R_L} = -\left(\frac{B}{\delta+B}\right)^2 e^{-\frac{L}{L_v}} \quad , \qquad (1)$$

where δ is proportional to the conductance of the metal contact. For simplicity, we assume that $1/\delta$ is scaled linearly with the magnetic field **B** based on the facts observed from the most conventional metals. Remarkably, the fitted curves perfectly match the experimental results as shown in Supplementary Fig. 9a. The calculated L_v adopts a similar temperature dependence as the one fitted from the length scaling but with a slightly smaller value. These similar results verify the calculated L_v.

Supplementary Note 7. A comparison of samples with different carrier density

Since the density of states for a 3D Dirac semimetal is proportional to E^2, the chiral magnetic effect from the charge pumping between Weyl nodes will be strongly suppressed with the increase of E_F. To give a direct comparison between samples with different carrier density, we list the transport behavior of two samples (Supplementary Table 1 and Supplementary Figs 11 and 12). Samples N1 and N3 were both grown by chemical vapor deposition but with slightly different conditions. We modified the growth condition of sample N1 by using arsenic power as extra source material, improving the vacuum, and shortening the time at high temperature. The resultant nanoplates show less vacancies and a lower Fermi level with the carrier density decreased by more than one order of magnitude at low temperature.

Similar nonlocal signals can be also reproduced from sample N3. However, the negative magnetoresistance is not observed and the nonlocal ratio α_{NL} is much smaller than the one shown in the main text. By performing the Hall effect and quantum oscillation measurements (Supplementary Fig. 11), we find that the carrier density and Fermi wave vector are relatively large in sample N3. Supplementary Fig. 12 summarizes a systematic comparison between these two samples. Supplementary Figs 12 a and b are the temperature-dependent resistance along different orientations. R_1 and R_2 correspond to the measurement geometries with in-plane current being parallel and perpendicular to [110] crystal direction, respectively. Consistent with bulk crystals, Cd_3As_2 nanoplates are anisotropic in conductance along different directions. The sample resistance increases when the temperature is reduced and becomes saturated below 10 K, sharing the same behavior as the lightly-doped Na_3Bi reported recently[1]. The increase of the resistance is consistent with the fact that the thermally-generated carriers are suppressed at low temperatures. The nonlocal signals are significantly reduced in sample N3 since the chiral anomaly is weakened due to the corresponding high Fermi level (Supplementary Figs 12 e and f). Meanwhile, the negative MR is also not observed at parallel **E**, **B** configuration (refer to Supplementary Fig. 12d). The close relationship between Fermi level and nonlocal response (or negative MR) highlights

the importance of high-quality sample with low carrier density for the detection of chiral anomaly. Meanwhile, the nonlocal transport serves as a much more sensitive probe than the negative MR based on the comparison of these experimental results.

Supplementary Note 8. Bulk Cd_3As_2 crystal characterizations

The as-grown single crystal Cd_3As_2 adopts {112} crystal planes (Supplementary Fig. 13a) with a large shining surface, which is perfect for obtaining a large reflection signal during the experiments. The crystal structure of our sample is found to be $I4_{1/acd}$ structure. It has a distorted superstructure of the antifluorite structure, whose unit cell is tetragonal with a=12.633(3) Å and c=25.427(7) Å. Each unit cell contains 96 Cd atoms and 64 As atoms. No ferromagnetic contamination is detected by the composition analysis (Supplementary Fig. 13b).

Supplementary Note 9. Magneto-optical Kerr effect (MOKE) of Cd_3As_2 bulk crystal

For comparison, we also performed ROT-MOKE measurements on the Cd_3As_2 bulk single crystal with the magnetic field fixed in the x-z plane. Two kinds of measurement configurations were adopted, in which the applied electric bias was parallel with and perpendicular to the x-z plane, respectively. In the former case, similar 360°-period ROT-MOKE curves were observed (Supplementary Fig. 14c), consistent with the angular dependence of $\mathbf{E} \cdot \mathbf{B}$ chiral gauge field. However, when the bias was added perpendicularly to the B-rotating plane, *i.e.* $\mathbf{E} \cdot \mathbf{B}$ became zero, no Kerr signal was detected (Supplementary Fig. 14d). These results can isolate the detected Kerr signals from the conventional magnetic order.

Supplementary Fig. 15 shows the relationship of Kerr rotation with magnetic field and current density. The current density is proportional to the electric field across the sample at a fixed temperature. As shown in Supplementary Fig. 15, the Kerr rotation is linearly scaled with the magnetic field and current density in the low current density regime. The consistency of electric and magnetic field dependence suggests a correlation effect of these two fields on the detected Kerr signal. Along with the angle dependence curve in the ROT-MOKE experiments, it suggests that the Kerr rotation is originated from the $\mathbf{E}\cdot\mathbf{B}$ field, a strong hint for the chiral anomaly. However, the deviation from the linear dependence is observed when further increasing the current density. It is still unclear why Kerr rotation deviates from linear dependence in the large current density regime. One possible reason is that the sample enters the regime of hot carrier transport under large current density where the electric field inside in the sample may acquire a different scaling law with the current density.

The MOKE experiments on the sample with electric bias were performed to explore the magnetic-field dependence of the Kerr signals. Firstly, we fixed the current density to be 144 mA mm^{-2} and swept the in-plane magnetic field back and forth at one certain direction in the y-z plane. The obtained MOKE signals show a quasi-linear relationship with the magnetic field but retain a distinct hysteresis during the sweeping

process (Supplementary Fig. 16). The quasi-linear behavior has no sign of saturation in our field range. The hysteresis is not affected by the sweeping speed or the sampling interval. By changing the sweeping field direction, we reproduced the angular-dependent Kerr rotation. The Kerr signals remain non-saturated with hysteresis which vanishes when the magnetic field is perpendicular to the electric field. Conventionally, the hysteresis in the Kerr signal is associated with ferromagnetic order. However, in our case, clearly no magnetic element is involved. The MOKE curve stays non-saturated at all angles in our field range of between -3000 and 3000 Oe, inconsistent with the scenario of spin flipping under external fields. The observed anomalous hysteresis is beyond the current understanding and requires further investigations.

Meanwhile we would like to mention a recent related work on Kerr effect in topological insulator Bi_2Te_2Se by Nirajan Mandal, *et al.*[21] In their study, current induced Kerr effect is observed. In contrast to our work, the Kerr signal in Bi_2Te_2Se does not require magnetic field. They attribute it to a linear birefringence effect and the current-induced spin- Kerr effect while the former one is dominant.

To trace the effect of temperature on the MOKE signals, we also carried out the Kerr experiments at different temperatures. As shown in Supplementary Fig. 17a, a similar $\mathbf{E} \cdot \mathbf{B}$ dependence was observed at 80 K. It was found that at a fixed current density, the Kerr rotation strength was not changed much from 80 K to 300 K (Supplementary Figs 17 b and c). To exclude the influence of sample resistance changing at different temperatures, we compare the Kerr rotation signals under the same voltage drop on the sample at different temperatures. The value of voltage can be precisely calculated by the measured current and the sample resistivity (4-probe) at the corresponding temperature. Only half of the Kerr rotation signal drop is witnessed from 80 K to 300 K, showing a robustness of the chiral charge pumping process (refer to Supplementary Fig. 17d).

Supplementary Note 10. Theoretical analysis on the mechanism of Kerr effect

The theory of circular dichroism in Weyl semimetals proposed by Hosur and Qi[10] is that the charge imbalance between the Weyl nodes can result in a non-zero gyrotropic coefficient γ, a Hall-like contribution to the dielectric tensor. Under the external $\mathbf{E}\cdot\mathbf{B}$ field, the corresponding chirality dependent chemical potential can be expressed as $\mu_\chi = \left(\mu^3 + \chi\frac{3e^2\hbar v_F^3}{2}\mathbf{E}\cdot\mathbf{B}\tau_v\right)^{1/3}$.[22] Here, μ is the initial chemical potential of Weyl nodes, v_F is the Fermi velocity and χ is the chirality of the Weyl nodes. For a single Weyl node, γ is given by: $\gamma(\omega) = i\frac{\chi\mu_\chi e^2\tau_{intra}}{6\pi^2\varepsilon_0\hbar^2\omega}$ with ω being the light frequency, τ_{intra} being the intra-valley scattering time. The real and imaginary parts of this Hall conductivity in gyrotropic tensor give the Faraday effect and circular dichroism, respectively. Note that the prerequisite of this theory is the low frequency limit ($\omega\tau_{intra} \ll 1$). Since the intra-node relaxation is very fast, the response stems from equilibrium configurations of each node, and we can only focus on the DC response. A single node preserves an effective time-reversal symmetry (TRS) but breaks effective inversion symmetry (IS) ("effective" means that the momentum is reversed at the location of the node). Under these symmetries, circular dichroism and Faraday rotation are allowed, but the Kerr effect vanishes. In addition, Faraday rotation vanishes in the low-frequency limit because it is proportional to the real part of the Hall conductivity, and a doped Weyl node in equilibrium has a zero net DC Hall conductivity. On the other hand, circular dichroism is proportional to the imaginary part of the Hall conductivity, which is non-zero.

However, if we go beyond the low frequency limit, *i.e.*, the intra-node relaxation is not fast enough, the response stems from non-equilibrium configurations of each Weyl node. Then a finite frequency electromagnetic response will emerge instead of the DC one. The Hall conductivity of each doped Weyl node is now complex. Thus, circular dichroism will certainly survive, and Faraday effect will appear. In addition, the Kerr effect will also be non-zero because the effective TRS is now broken. All these effects will be proportional to $\mathbf{E}\cdot\mathbf{B}$ because they all come from the chiral charge

pumping process due to the chiral anomaly. The theoretical analysis on the non-equilibrium configurations is attributed to the valuable discussions with Dr. Pavan Hosur. The initial assumption on the low frequency limit can be confirmed by the transport results as presented in the main text.

Supplementary Note 11. Electrical transport properties of Cd_3As_2 bulk crystal

Standard magnetoresistance measurements were conducted to probe the transport properties of Cd_3As_2 single crystal. A constant current was applied within the (112) atomic planes while the magnetic field was titled from perpendicular to parallel to the (112) planes. Strong anisotropy MR and quantum oscillations were clear resolved at low temperatures as shown in Supplementary Fig. 18. The ρ_{xx}-T curve describes a typical metallic behavior owing to the semimetal band structure, consistent with previous reports[14]. No negative longitudinal MR is observed owing to the large MR and relatively high Fermi level.

Supplementary References

1. Xiong, J., *et al.* Evidence for the chiral anomaly in the Dirac semimetal Na_3Bi. *Science* **350**, 413-416 (2015).
2. Li, Q., *et al.* Chiral magnetic effect in $ZrTe_5$. *Nat. Phys.* **12**, 550–554 (2016).
3. Huang, X., *et al.* Observation of the chiral anomaly induced negative magneto-resistance in 3D Weyl semi-metal TaAs. *Phys. Rev. X* **5**, 031023 (2015).
4. Zhang, C.-L., *et al.* Signatures of the Adler-Bell-Jackiw chiral anomaly in a Weyl fermion semimetal. *Nat. Commun.* **7**, 10735 (2015).
5. Yang, X., Liu, Y., Wang, Z., Zheng, Y., Xu, Z.-a. Chiral anomaly induced negative magnetoresistance in topological Weyl semimetal NbAs. *Preprint at http://arxiv.org/abs/1506.03190* (2015).
6. Hirschberger, M., *et al.* The chiral anomaly and thermopower of Weyl fermions in the half-Heusler GdPtBi. *Nat. Mater.* **Advance Online Publication**, (2016).
7. Li, C.-Z., Wang, L.-X., Liu, H., Wang, J., Liao, Z.-M., Yu, D.-P. Giant negative magnetoresistance induced by the chiral anomaly in individual Cd_3As_2 nanowires. *Nat. Commun.* **6**, 10137 (2015).
8. Li, H., *et al.* Negative Magnetoresistance in Dirac Semimetal Cd_3As_2. *Nat. Commun.* **7**, 10301 (2015).
9. Parameswaran, S., Grover, T., Abanin, D., Pesin, D., Vishwanath, A. Probing the chiral anomaly with nonlocal transport in three-dimensional topological semimetals. *Phys. Rev. X* **4**, 031035 (2014).
10. Hosur, P., Qi, X.-L. Tunable circular dichroism due to the chiral anomaly in Weyl semimetals. *Phys. Rev. B* **91**, 081106 (2015).
11. Lee, P. A., Ramakrishnan, T. Disordered electronic systems. *Rev. Mod. Phys.* **57**, 287 (1985).
12. Lee, P. A., Fisher, D. S. Anderson localization in two dimensions. *Phys. Rev. Lett.* **47**, 882 (1981).
13. Jeon, S., *et al.* Landau quantization and quasiparticle interference in the three-dimensional Dirac semimetal Cd_3As_2. *Nat. Mater.* **13**, 851-856 (2014).
14. Liang, T., Gibson, Q., Ali, M. N., Liu, M., Cava, R. J., Ong, N. P. Ultrahigh mobility and giant magnetoresistance in the Dirac semimetal Cd_3As_2. *Nat. Mater.* **14**, 280-284 (2014).
15. Cao, J., *et al.* Landau level splitting in Cd_3As_2 under high magnetic fields. *Nat. Commun.* **6**, 7779 (2015).
16. Hu, J., Rosenbaum, T., Betts, J. Current jets, disorder, and linear magnetoresistance in the silver chalcogenides. *Phys. Rev. Lett.* **95**, 186603 (2005).
17. Luo, Y., *et al.* Anomalous magnetoresistance in $TaAs_2$. *Preprint at http://arxiv.org/abs/1601.05524* (2016).
18. Arnold, F., *et al.* Negative magnetoresistance without well-defined chirality in the Weyl semimetal TaP. *Nat. Commun.* **7**, 11615 (2016).
19. Abanin, D., *et al.* Giant nonlocality near the Dirac point in graphene. *Science* **332**, 328-330 (2011).

20. Gorbachev, R., *et al.* Detecting topological currents in graphene superlattices. *Science* **346**, 448-451 (2014).
21. Mandal, N., Mitkowski, I., Glazov, M., Chen, Y. Current induced Optical Activity in Topological Insulator Bi2Te2Se1. *APS Meeting Abstracts* (2016).
22. Zhou, J., Chang, H.-R., Xiao, D. Plasmon mode as a detection of the chiral anomaly in Weyl semimetals. *Physical Review B* **91**, 035114 (2015).
